# A multi-omics case–control study identifying oropharyngeal microbiome–metabolite patterns that characterize secondary bacterial pneumonia among influenza patients

**DOI:** 10.3389/fmicb.2026.1824965

**Published:** 2026-05-22

**Authors:** Hong Zhang, Ran He, Lei Xu, Hang Zhou, Rongbin Yu, Peng Huang

**Affiliations:** Key Laboratory of Public Health Safety and Emergency Prevention and Control Technology of Higher Education Institutions in Jiangsu Province, National Vaccine Innovation Platform, Department of Epidemiology, Center for Global Health, School of Public Health, Nanjing Medical University, Nanjing, China

**Keywords:** 16S rRNA, machine learning, oropharyngeal microbiome, secondary pneumonia, untargeted plasma metabolomics

## Abstract

Secondary bacterial pneumonia is a severe complication of influenza;howeve the biological determinants that distinguish progression from uncomplicated infection remain poorly understood. We investigated the oropharyngeal microbiome and plasma metabolome as potential discriminators of pneumonia development. In this study, we report a cross-sectional case–control study conducted during the 2022–2023 influenza season to identify and internally validate a microbiome-metabolite profile that characterizes pneumonia cases from uncomplicated influenza. We enrolled 236 consecutive influenza patients from Jiangsu Province, China (October 2023–December 2024): 59 with secondary pneumonia and 177 uncomplicated controls. Oropharyngeal swabs were subjected to 16S rRNA V3-V4 sequencing; plasma metabolomics was performed by UPLC-MS/MS in both ion modes. Seven machine-learning algorithms were compared; Least Absolute Shrinkage and Selection Operator (LASSO) logistic regression was selected because it yielded the highest cross-validated discrimination. Microbial composition distinguished groups, not richness. Pneumonia cases showed enrichment of *Synergistota* and *Bifidobacteriaceae* with depletion of *Bacillaceae* (β-diversity *p* = 0.057). Controls exhibited enriched glycolysis and lipid metabolism pathways; pneumonia cases showed elevated degradation pathways (GLUCARDEG and GALLATE-DEGRADATION). Plasma metabolomics revealed a lipid depletion signature: phospholipids PC(O-16:0/0:0) and PS(14:0/18:3(9Z,12Z,15Z)) were significantly reduced (area under the (receiver operating characteristic) curves (AUCs) = 0.69–0.71). Small Molecule Pathway Database (SMPDB) pathway analysis demonstrated suppressed anabolic (tyrosine, steroid, and purine metabolism) and enhanced catabolic (beta-oxidation of very long-chain fatty acids) pathways. Machine learning identified *Peptococcus* as the top indicator (LASSO AUC = 0.65); Shapley Additive Explanation (SHAP) analysis revealed a monotonic risk increase with abundance. Oropharyngeal dysbiosis and systemic metabolic reprogramming characterize influenza cases that progress to secondary pneumonia. *Peptococcus* and four metabolites form an internally validated exploratory profile associated with secondary pneumonia; external validation and performance optimization are warranted.

## 1 Introduction

Influenza remains a major global health threat (Goeijenbier et al., 2017; Hanada et al., 2018; Kalil and Thomas, 2019; Kramer et al., 2024; Laurichesse et al., 2023; Morris et al., 2017; Wolter et al., 2014), with secondary bacterial pneumonia representing a leading cause of mortality and intensive care unit (ICU) admission (Rothberg et al., 2008; Smith and McCullers, 2014; Lu et al., 2017). Despite advances in antiviral therapy (Kumari et al., 2023), no reliable biomarkers can currently distinguish influenza patients who will progress to pneumonia, limiting targeted interventions. The pathophysiology underlying this progression remains poorly understood, particularly the role of the oropharyngeal microbiome as a gatekeeper of lower respiratory health (Faner et al., 2017; Ren et al., 2025).

The upper respiratory tract harbors a complex microbial community (Shilts et al., 2022; Nath et al., 2023; Perdijk et al., 2023) that regulates local immunity (Sanches Santos Rizzo Zuttion et al., 2024) and prevents pathogen colonization (Lindgren et al., 2023). The oropharynx represents a critical ecological gateway to the lower respiratory tract. Microaspiration of oropharyngeal secretions is the primary mechanism of lower respiratory tract colonization and infection, particularly in the context of viral-induced epithelial damage and impaired mucociliary clearance. In influenza, oropharyngeal dysbiosis has been associated with increased bacterial burden and cytokine dysregulation (Hu et al., 2016). In the specific context of secondary bacterial pneumonia following influenza, the oropharynx assumes particular relevance for several reasons: Microaspiration is the predominant route of bacterial entry into the lungs in community-acquired pneumonia; influenza specifically compromises upper respiratory defense mechanisms—mucociliary clearance, epithelial barrier integrity, and local immune surveillance—creating a conduit for oropharyngeal bacterial dissemination; practical and ethical constraints preclude routine bronchoscopic sampling in outpatient influenza patients with uncomplicated disease, and oropharyngeal swabs offer a non-invasive, feasible surrogate that captures the microbial reservoir available for aspiration. However, no study has linked taxonomic shifts to the development of secondary pneumonia, nor have functional analyses been integrated to infer underlying mechanisms.

Microbial metabolites in the oropharynx can enter systemic circulation (Peng et al., 2022; Xu et al., 2025), reflecting host–pathogen interactions. Previous metabolomics studies in sepsis and pneumonia have identified altered bile acids, phospholipids, and amino acid derivatives as severity markers (Chen et al., 2022). However, no investigation has simultaneously characterized the oropharyngeal microbiome and plasma metabolome in influenza patients to determine whether microbial function drives systemic metabolic dysregulation before pneumonia.

Integrating high-dimensional microbiome, metabolome, and clinical data poses analytical challenges that conventional statistics cannot resolve. Machine learning algorithms excel at identifying non-linear interactions (Orsini et al., 2022) and prioritizing discriminative features across disparate data types. We hypothesized that an integrated multi-omics model would outperform single-modality approaches and reveal discriminative microbiome–metabolome axes in influenza patients.

In this study, we leveraged a case–control study of 236 influenza patients (59 with secondary pneumonia), performing 16S rRNA sequencing of oropharyngeal swabs (Koenen et al., 2023), untargeted plasma metabolomics in both ion modes (Fryk et al., 2024), and machine learning integration. Our clinical data and rigorous covariate adjustment enabled us to identify specific microbial taxa, functional pathways, and plasma metabolites associated with pneumonia progression. We demonstrate that *Peptococcus*, *Anoxybacillus*, and 5-sulfosalicylic acid are top-ranked discriminative features, providing a foundation for microbiome-directed interventions in influenza.

To date, no case–control study has integrated simultaneous oropharyngeal microbiome and plasma metabolome profiling to derive a profile for secondary bacterial pneumonia in influenza outpatients.

## 2 Methods

### 2.1 Experimental model and study participant details

Study participants were recruited from outpatients and inpatients presenting to the Infectious Disease Fever Clinic or wards at a regional hospital in Jiangsu Province between October 2022 and December 2023. Clinical data collection was performed at the time of outpatient presentation and was limited by the acute care setting. Available variables included demographic characteristics [age, sex, and body mass index (BMI)], basic vital signs (maximum body temperature), and routine blood chemistry [complete blood count and C-reactive protein (CRP)]. Prior to enrollment, all participants received comprehensive information regarding the study’s objectives, procedures, required participation, anticipated benefits, and potential risks. Upon confirming their understanding that participation was voluntary and that they could refuse or withdraw at any time, participants provided written informed consent. The inclusion criteria were provision of informed consent, voluntary participation, and age between 18 and 80 years. The exclusion criteria were missing oropharyngeal swabs or blood samples or the absence of baseline demographic data (age and sex). The study design and implementation procedures involving human participants were approved by the Ethics Committee of Nanjing Medical University and conducted in accordance with the 1975 Declaration of Helsinki, as revised in 2008. All protocols were approved by the Institutional Review Board of Nanjing Medical University (IRB Protocol: (2021)519), and informed consent was obtained from every study subject.

### 2.2 Pneumonia diagnosis criteria

Influenza patients were defined according to the *Influenza Diagnosis and Treatment Protocol (2020 Revised Edition)* issued by China’s National Health and Family Planning Commission as individuals presenting with influenza-associated clinical symptoms (fever, cough, rhinitis, and respiratory distress). Patients with secondary pneumonia were identified by applying the *Chinese Guidelines for the Diagnosis and Treatment of Adult Community-Acquired Pneumonia (2018 edition)* to chest radiography findings, and we consecutively enrolled 387 influenza patients. Among these patients, 59 patients developed secondary bacterial pneumonia. The remaining 328 patients had uncomplicated influenza and served as the pool for control selection. To construct a balanced case–control sample for multi-omics analysis, we applied propensity score matching (PSM) to select controls from the 328 eligible patients. Propensity score matching protocol: We constructed propensity scores using a logistic regression analysis with secondary pneumonia as the outcome. Matching variables included age, sex, and clinical symptoms to control for demographic factors and seasonal variation. Matching was performed using nearest neighbor matching with a caliper of 0.2 standard deviations of the logit-transformed propensity score, without replacement, at a 1:3 ratio. This procedure yielded 177 matched controls.

### 2.3 Microbiome processing and analysis

Oropharyngeal swabs were collected using standardized sterile nylon-flocked swabs (Copan Diagnostics, Murrieta, CA) after a 30-min fasting period with a water rinse to minimize food contamination. Collection targeted the bilateral tonsillar fossae and posterior pharyngeal wall, with the swab rotated three to five times against mucosal surfaces while avoiding contact with teeth, tongue, and gingiva. Swabs were immediately placed in 1 mL sterile phosphate-buffered saline (PBS), transported on ice, and frozen at 4–8 °C within 4 h of collection. This protocol captures the oropharyngeal microbiome while minimizing contamination from the oral cavity (buccal mucosa and tongue) and nasopharyngeal sources. DNA was extracted using the QIAamp Fast DNA Mini Kit (Qiagen Venlo, the Netherlands). 16S V3-V4 amplicons were generated with primers 338F/806R and sequenced on MiSeq (TruSeq kit San Diego, California, USA). QIIME 2 (v2023.7) DADA2 workflow produced ASVs; taxonomy was assigned against SILVA via the sklearn classifier (Bolyen et al., 2019). Alpha diversity (Kruskal–Wallis) and beta diversity [permutational multivariate analysis of variance (PERMANOVA)] were computed in phyloseq (R v4.3.0). Differential taxa were identified by linear discriminant analysis effect size (LEfSe) (linear discriminant analysis [LDA] > 4.0) after filtering features present in <5% of samples or <0.1% relative abundance.

### 2.4 Metabolomics

Plasma metabolites were extracted using a protein precipitation protocol. Specifically, 100 μL plasma was mixed with 400 μL ice-cold 80% methanol (v/v in water) to precipitate proteins and solubilize metabolites. The mixture was vortexed for 30 s, incubated at −20 °C for 30 min, and then centrifuged at 12,000 g for 15 min at 4 °C. The resulting supernatant containing soluble plasma metabolites was transferred to a clean tube, evaporated to dryness under nitrogen, and reconstituted in 100 μL 80% methanol for UPLC-MS/MS analysis. This extraction targets small-molecule metabolites (amino acids, lipids, organic acids, and nucleotides) rather than plasma proteins or macromolecules. UPLC-MS/MS conditions: Chromatographic separation was performed on an ACQUITY UPLC BEH C18 column (1.7 μm, 2.1 × 100 mm; Waters) with mobile phases A (0.1% formic acid in water) and B (0.1% formic acid in acetonitrile). The gradient program was as follows: 0–2 min 5%B, 2–20 min 5–95% B, 20–22 min 95% B, 22–22.1 min 95–5%B, 22.1–25 min 5% B. The flow rate was 0.4 mL/min; the column temperature was 4 °C; the injection volume was 5 μL. Mass spectrometry was performed in both positive and negative electrospray ionization modes with data-dependent MS/MS acquisition (Top3). The source parameters were as follows: capillary voltage 2.0 kV (positive)/1.5 kV (negative), desolvation temperature 500 °C, and source temperature 150 °C.

Quality control (QC) samples were prepared by pooling equal volumes (10 μL) from each study plasma sample (*n* = 236), creating a representative composite sample. This QC pool was processed identically to study samples and analyzed repeatedly throughout the run sequence—specifically, at the beginning of the batch, after every 5–15 study samples, and at the end of the run. QC samples served three purposes: (i) system suitability assessment (retention time stability and mass accuracy), (ii) data quality monitoring (coefficient of variation <20% for detected features), and (iii) batch effect correction if drift exceeded pre-specified thresholds. Features with >30% missing values or CV > 30% in QC samples were excluded from downstream analysis.

### 2.5 Multi-omics machine-learning model building

We built integrated discrimination models in R 4.3.0. To address the high dimensionality of the multi-omics data (approximately 1,200 features from 236 samples) and to minimize overfitting, we implemented a rigorous three-stage feature reduction strategy: (i) Microbiome features were pre-filtered using linear discriminant analysis effect size (LEfSe) with an LDA score of >3.0, and metabolome features were selected by partial least squares discriminant analysis (PLS-DA) variable importance in projection (VIP) of >1.0. This reduced the feature space from ~1,200 to 193 candidate variables. To address the residual age imbalance after PSM, age was included as a forced covariate in all machine learning models. Within the same case–control sample, we randomly split participants into a 70% internal derivation set (training/validation) and a 30% internal hold-out fold for final performance estimation; both subsets maintained the 1:3 case-to-control ratio. (ii) The following seven algorithms were trained on the derivation set: Random Forest (RF, mtry = 8,999 √p, 500 trees); Gradient Boosting Machine (GBM, shrinkage = 0.01, interaction depth = 3); Generalized Linear Model (GLM, logistic); k-Nearest Neighbors (KNN, *k* = 5, triangular kernel); Neural Network (NNET, single hidden layer, size = 3, decay = 0.01); Least Absolute Shrinkage and Selection Operator (LASSO, *α* = 1; λ by 1-SE rule); Decision Tree (DT, min-split = 5, complexity parameter = 0.01). (iii) Model performance was evaluated using 5-fold repeated cross-validation with class weights to address imbalance. Hyperparameter tuning was performed within the inner CV loop via random search (50 iterations) to prevent information leakage. Discrimination was quantified by area under the receiver operating characteristic curve (ROC-AUC) with 2,000 bootstrap CIs; calibration and decision-curve analyses were omitted because case–control sampling does not support unbiased probability estimation. Variable importance was ranked by mean root mean square error (RMSE) loss after 50 permutations; SHAP dependence plots confirmed monotonicity of the top discriminator. LASSO achieved the highest cross-validated ROC-AUC and was retained as the final discrimination profile.

### 2.6 Bioinformatics tools

Microbiome analysis was performed using QIIME 2 (v2023.7). Plasma metabolome analysis was partially conducted on the MetaboAnalyst online platform (Pang et al., 2024). R scripts were executed in R (v4.3.0).

### 2.7 Quantification and statistical analysis

Continuous variables are presented as mean±SD or median (IQR) and categorical variables as *n* (%). For group comparisons, Student’s *t*-test or the Mann–Whitney U test was used for continuous variables, and the χ^2^ test or Fisher’s exact test was used for categorical variables. Statistical significance was assessed at *α* = 0.05 unless otherwise specified. For multiple comparisons, we applied the following correction strategies: Microbiome differential abundance (LEfSe): LDA scores of >4.0 were considered significant; no formal multiple testing correction was applied due to the exploratory nature, but features were filtered to require presence in >5% of samples with >0.1% relative abundance to reduce false positives. Benjamini-Hochberg false discovery rate (FDR) correction was applied for metabolite comparisons; metabolites with FDR-adjusted *p*-value < 0.05 were reported as significant. Machine learning: There was no multiple testing correction for model comparison, as these were descriptive performance estimates with confidence intervals.

## 3 Results

### 3.1 Oropharyngeal microbiome profile characterizes influenza patients with secondary bacterial pneumonia

**Figure 1:**
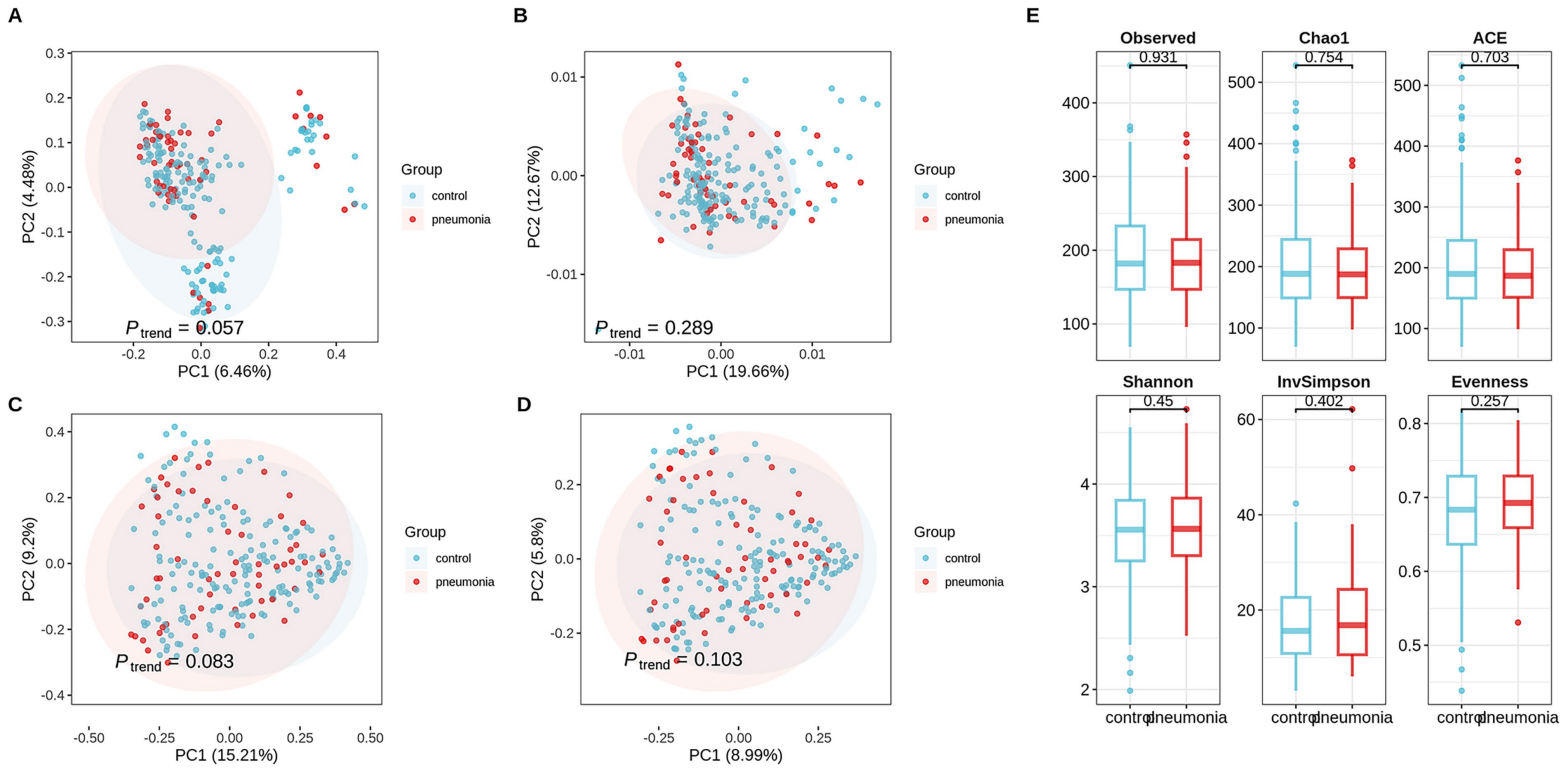
Oropharyngeal microbiota alpha and beta diversities differ between influenza patients with and without secondary pneumonia. Principal coordinate analysis (PCoA) based on unweighted UniFrac distances (PC1 = 6.46%, PC2 = 4.48%) **(A)**. PCoA based on weighted UniFrac distances (PC1 = 19.66%, PC2 = 12.67%) **(B)**. PCoA using Bray–Curtis dissimilarity (PC1 = 15.21%, *P* trend = 0.083, PC2 = 9.2%) **(C)**. PCoA using Jaccard distance (PC1 = 8.99%, *P* trend = 0.103, PC2 = 5.8%) **(D)**. Alpha diversity comparisons showing observed richness, Chao1, and ACE estimates **(E)**. *n* = 59 influenza patients with secondary pneumonia and *n* = 177 influenza-only controls; full demographic and clinical characteristics are provided in Supplementary Table S1. Data representation: Box plots in **(E)** denote median, interquartile range, and whiskers extending to 1.5 × IQR. Each point in PCoA plots **(A–D)** represents a single oropharyngeal swab sample, colored by group.

To identify microbiome features that characterize influenza patients who develop secondary bacterial pneumonia, we compared oropharyngeal swabs from 59 influenza patients with pneumonia and 177 uncomplicated controls. Age differed significantly between groups (45.66 ± 15.70 versus 39.70 ± 17.18 years, *p* < 0.001), whereas remaining demographics were similar (Supplementary Table S1). This difference reflects the strong association between older age and pneumonia risk, which was incompletely addressed by matching with caliper constraints. Analytical adjustment for age in downstream models was implemented to address this limitation. 16S rRNA V3-V4 sequencing yielded a median of 4,250 reads per sample, with *Firmicutes* and *Bacteroidota* dominating both groups (Supplementary Figures S1B,C). LEfSe identified taxa enriched in cases: *Synergistaceae*, *Synergistales*, *Synergistia*, *Bifidobacteriales*, and *Bifidobacteriaceae*; controls showed higher relative abundances of *Bacillaceae* and *Bacillales* (Supplementary Figure S1D). β-diversity differed by pneumonia status: Unweighted UniFrac exhibited the strongest separation (*p* = 0.057; Figure 1A). We acknowledge that this does not reach conventional statistical significance (α = 0.05) and should be interpreted as a suggestive trend requiring confirmation. Weighted UniFrac, Bray–Curtis, and Jaccard distances showed similar trends (Figures 1B–D). α-diversity metrics (observed richness, Chao1, and ACE) did not differ significantly (*p* > 0.05; Figure 1E), indicating that community composition, rather than richness, characterizes cases versus controls within this case–control sample.

### 3.2 Discriminative microbial taxa in secondary pneumonia

**Figure 2:**
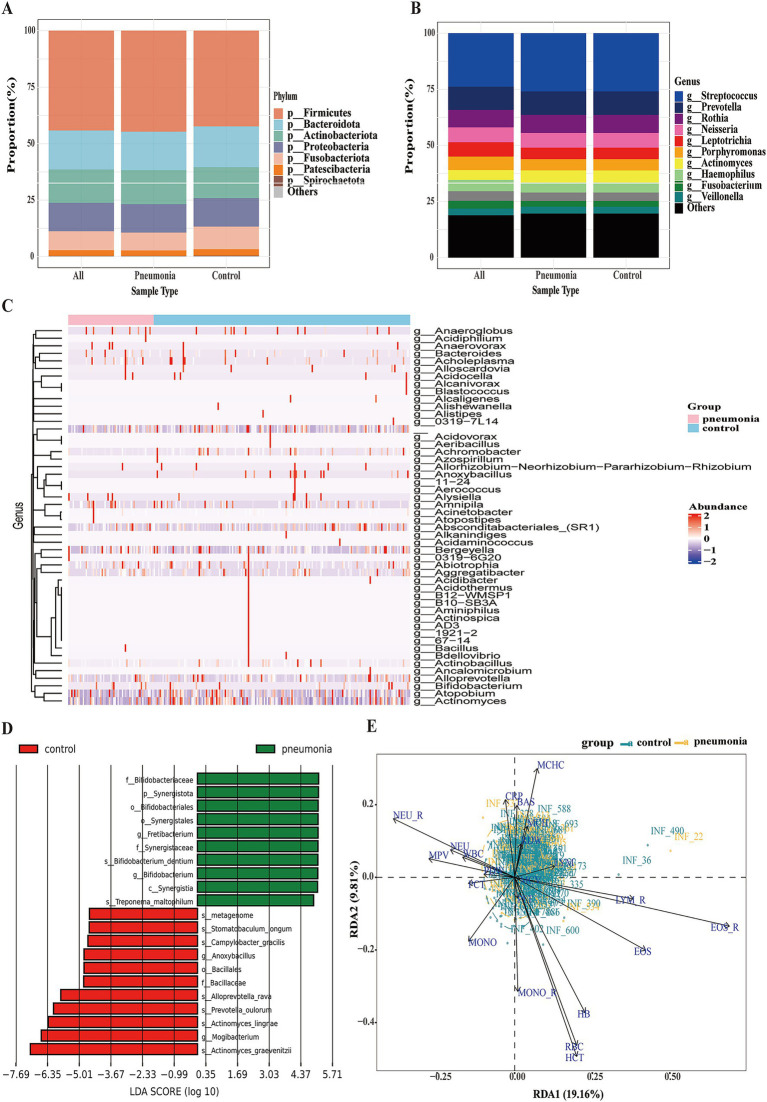
Oropharyngeal microbiome composition and discriminative taxa in influenza patients with versus without secondary pneumonia. Phylum-level taxonomic composition shown as stacked bars for each group **(A)**. Genus-level taxonomic composition displayed as stacked bars **(B)**. Heatmap of the top 50 most abundant genera, with rows z-scored and hierarchically clustered **(C)**. LEfSe cladogram identifying discriminative taxa between control (influenza alone) and pneumonia (influenza with pneumonia) groups; taxa with LDA score are shown **(D)**. Redundancy analysis (RDA) biplot of microbial community constrained by blood biochemical parameters (RDA1 = 19.16%, RDA2 = 9.81%) **(E)**. Data representation: Stacked bars in **(A,B)** mean relative abundance per group. Heatmap **(C)** displays z-scored relative abundances with dendrogram clustering.

To identify specific taxa driving compositional differences, we analyzed microbial profiles at multiple taxonomic levels. Phylum-level abundance of *Firmicutes* and *Bacteroidota* did not differ between groups (Figure 2A). While *Streptococcus* and *Prevotella* dominated at the genus level, their proportions varied between cases and controls (Figure 2B). Hierarchical clustering of the top 50 genera revealed distinct group-specific profiles (Figure 2C). LEfSe identified *Bifidobacteriaceae* and *Synergistota* enrichment in pneumonia cases (LDA ≈ 4.54), whereas *Mogibacterium* and *Actinomyces lingrae* were enriched in controls (Figure 2D).

We next asked whether systemic inflammation underpins these microbial differences. Constrained analysis revealed that blood markers explained 29.0% of microbial variation (RDA1 = 19.16%, RDA2 = 9.81%; Figure 2E). Absolute neutrophil count (NEU) percentage and MCHC correlated with pneumonia-enriched taxa, while absolute neutrophil count (LYM) and EOS percentages associated with control taxa. Consistently, pneumonia cases exhibited elevated WBC, NEU_R, MONO_R, MCHC, and CRP (*p* < 0.05), with BAS_R and NEU ratios reaching a *p*-value < 0.01 (Supplementary Table S2). These findings link systemic inflammation to oropharyngeal dysbiosis within this case–control sample.

### 3.3 Functional microbiome pathways correlate with pneumonia status

**Figure 3:**
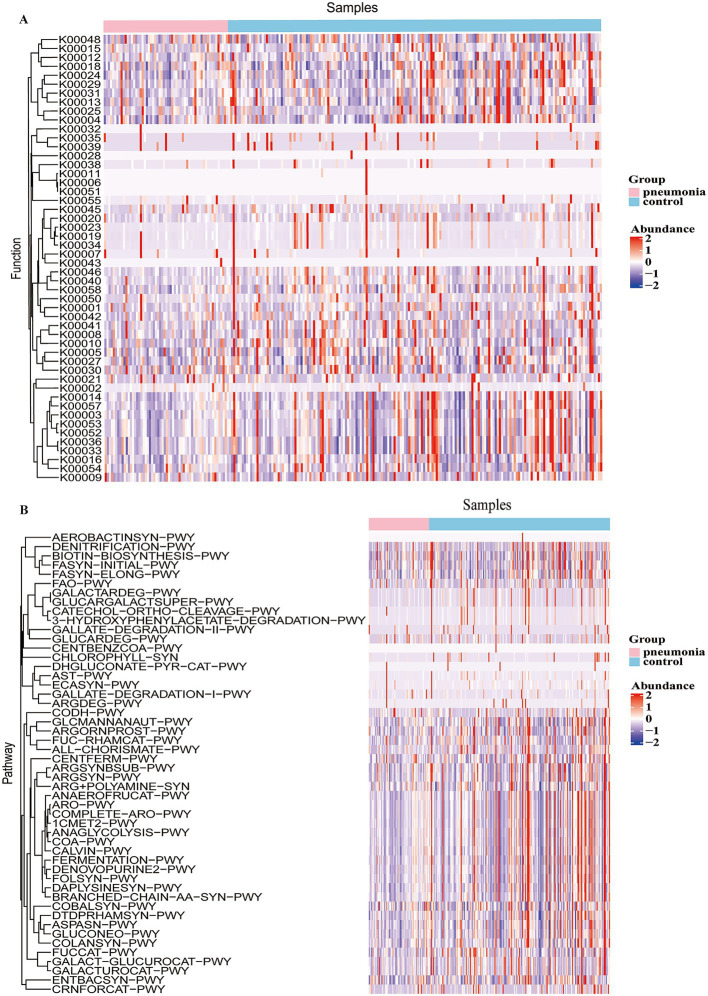
Heatmaps of functional gene and pathway enrichment in the oropharyngeal microbiome of influenza patients with and without secondary pneumonia. A heatmap of the 50 most discriminative gene families predicted by PICRUSt2 **(A)**. A heatmap of the 50 most discriminative metabolic pathways between control (influenza alone) and pneumonia (influenza with secondary pneumonia) groups **(B)**. Data representation: Heatmaps display row-z-scaled relative abundances with hierarchical clustering by Euclidean distance. Rows represent gene families **(A)** or pathways **(B)**; columns represent individual samples grouped by clinical status.

We generated KEGG Orthology (KO) and MetaCyc pathway abundance discriminations. Hierarchical clustering of the 50 most discriminative KOs (by eBayes) revealed control-enriched KOs involved in glycolysis, pentose phosphate, and lipid metabolism; pneumonia-specific enrichment was less pronounced (Figure 3A). Microbial functional pathways were inferred using PICRUSt2 based on 16S rRNA gene sequences. These are computational predictions of metagenomic content and should be interpreted as hypotheses rather than direct measurements of gene expression or metabolic flux. We observed predicted enrichment of degradation pathways (GLUCARDEG-PWY, GALLATE-DEGRADATION-I-PWY, and ARG + POLYAMIINE-SYN) in pneumonia cases and predicted enrichment of glycolysis, whereas controls were enriched for GALACTARDEG-PWY, GLUCARGALACTSUPER-PWY, CATECHOL-ORTHO-CLEAVAGE-PWY, and eight additional pathways (Figure 3B).

**Figure 4:**
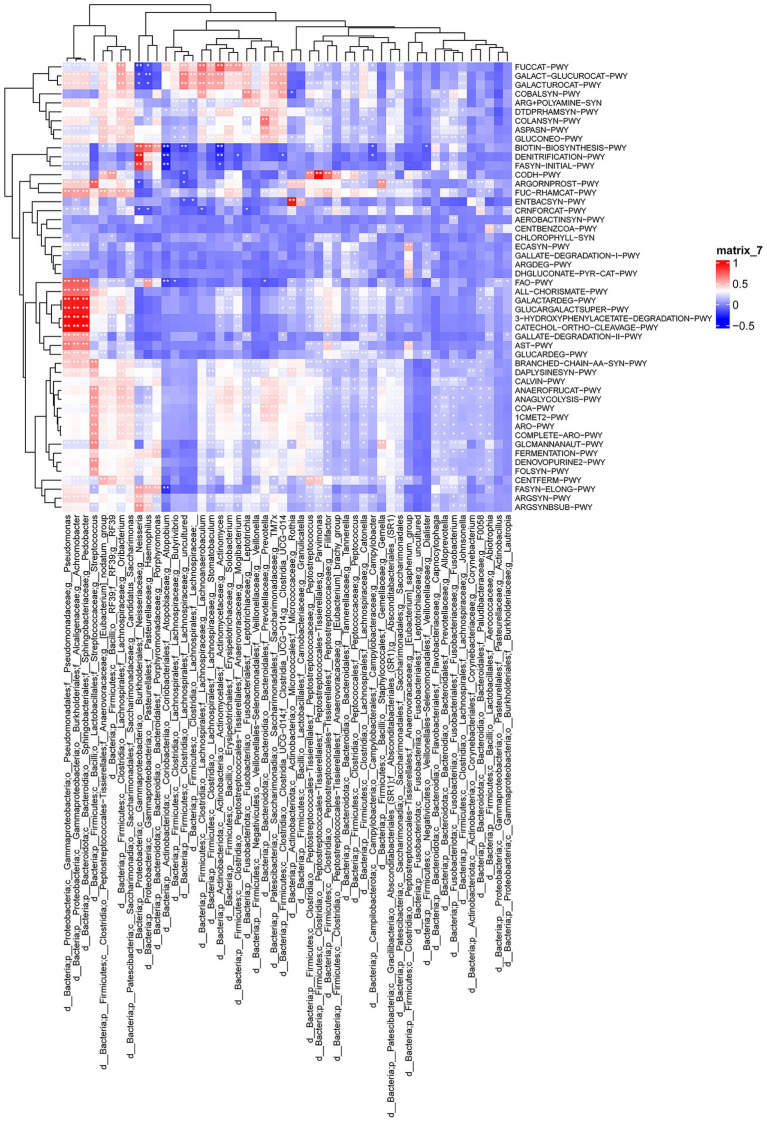
Correlation analysis links differentially abundant oropharyngeal microbiota with functional pathways in influenza patients with versus without secondary pneumonia. Spearman’s correlation heatmap between the top 50 differentially abundant microbial genera and top 50 differentially abundant KEGG pathways between control (influenza alone) and pneumonia (influenza with secondary pneumonia) groups. Significance: *0.01 < *p* < 0.05, **p < 0.01. Data representation: The heatmap displays Spearman’s correlation coefficients (r), with colors indicating correlation strength and direction (red = positive and blue = negative). Row and column dendrograms represent hierarchical clustering.

To link taxa to function, we performed Spearman’s correlation analysis between the top 50 differential genera and KO pathways. *Pseudomonas*, *Achromobacter*, and *Pedobacter* correlated strongly with aromatic compound degradation pathways (GALACTARDEG-PWY, GLUCARGALACTSUPER-PWY, 3-HYDROXYPHENYLACETATE-DEGRADATION-PWY, and CATECHOL-ORTHO-CLEAVAGE-PWY). *Neisseria* correlated with denitrification (DENITRIFICATION-PWY) and fatty acid synthesis (FASYN-INITIAL-PWY). Additional significant correlations included *Parvimonas* with CODH-PWY, *Rothia* with ENTBACSYN-PWY, and *Prevotella* with COLANSYN-PWY (*p* < 0.05; Figure 4). These taxa–pathway interactions suggest functional dysbiosis in the oropharyngeal microbiome characterizes pneumonia cases from controls within this case–control sample.

### 3.4 Plasma metabolites in both ion modes characterize influenza patients with secondary pneumonia

**Figure 5:**
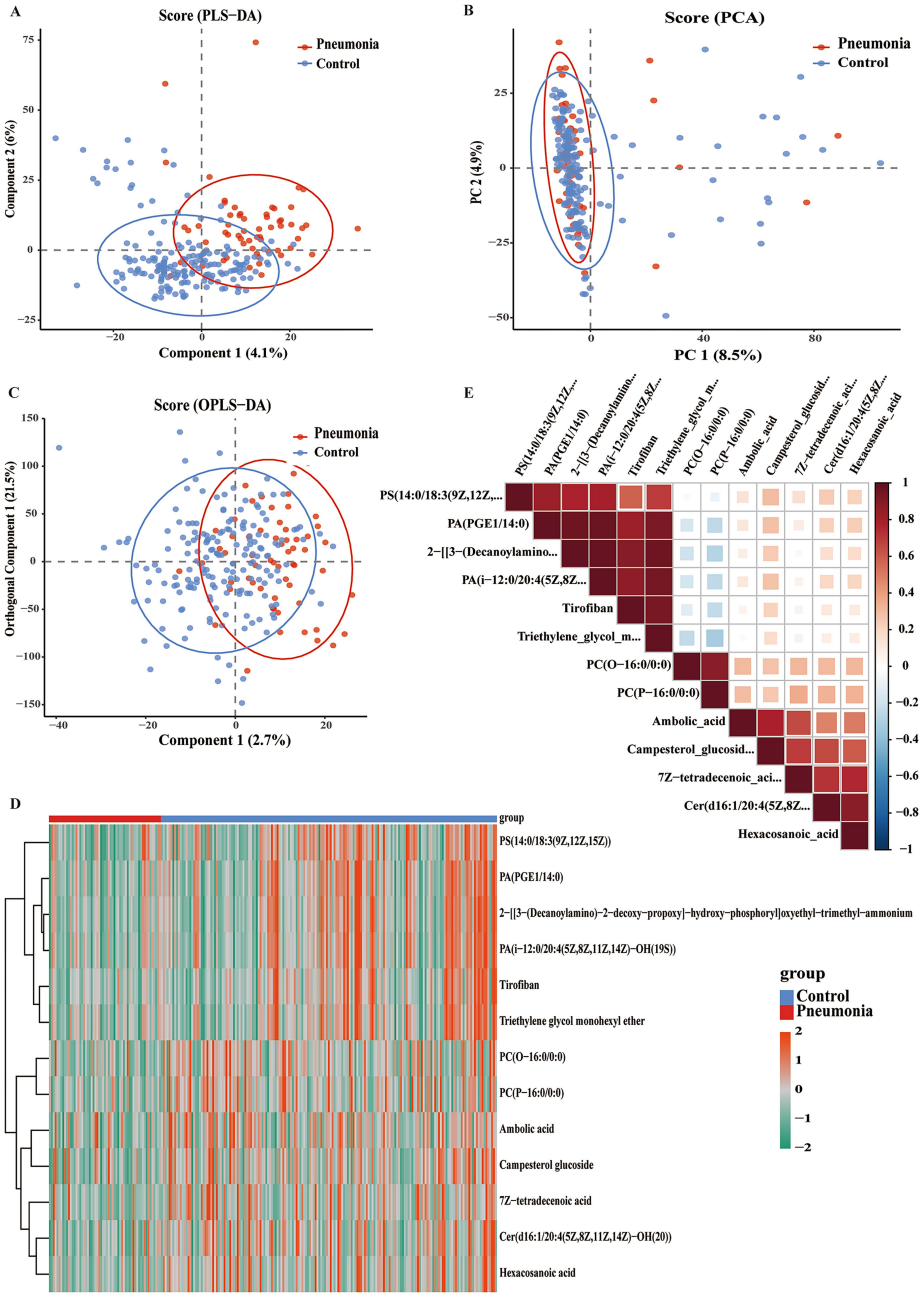
Distribution of integrated plasma metabolomes in positive and negative ion modes in influenza patients with and without secondary pneumonia. PLS-DA score plot separating control (influenza alone) versus pneumonia (influenza with secondary pneumonia) groups **(A)**. PCA score plot of combined ion mode data **(B)**. OPLS-DA score plot separating control (influenza alone) versus pneumonia (influenza with secondary pneumonia) groups **(C)**. Heatmap of differential metabolites (cutoff > 0, *p* < 0.05) with hierarchical clustering **(D)**. Spearman correlation heatmap of differential metabolites shown in **(E)**. Data representation: PCA, PLS-DA, and OPLS-DA plots show 95% confidence ellipses. Heatmap **(D)** is filtered by VIP from OPLS_DA model. Correlation heatmap **(E)** uses color gradient for correlation strength (red = positive, blue = negative).

To define the systemic metabolic profile that discriminates influenza patients with secondary bacterial pneumonia from uncomplicated controls, we performed plasma metabolomics in both ion modes. Multivariate modeling achieved clear separation: PCA (PC1 = 8.5%; Figure 5B) and PLS-DA (Component 1 = 4.1%, Component 2 = 6%; Figure 5A) distinguished pneumonia from controls, with OPLS-DA further improving resolution (Figure 5C). VIP filtering identified 12 differential metabolites that clustered into pneumonia- and control-specific modules (Figure 5D). Spearman’s correlation analysis revealed two functional modules: phospholipids (PC(O-16:0/0:0), PC(P-16:0/0:0)), and Cer(d16:1/20:4(5Z,8Z.)) formed a positively correlated cluster (*r* > 0.6), while xenobiotics (tirofiban) and long-chain fatty acids (hexacosanoic acid, *r* < −0.5) were strongly negatively correlated. Prostaglandin-bound phospholipids PS(14:0/18:3(9Z,12Z,15Z)) and PA(PGE1/14:0) correlated positively with each other but negatively with saturated fatty acids (Figure 5E). These findings demonstrate that phospholipid remodeling is inversely associated with xenobiotic metabolism and fatty acid elongation within this case–control sample.

### 3.5 Plasma metabolite biomarkers and SMPDB pathway enrichment characterize influenza patients with secondary pneumonia

**Figure 6:**
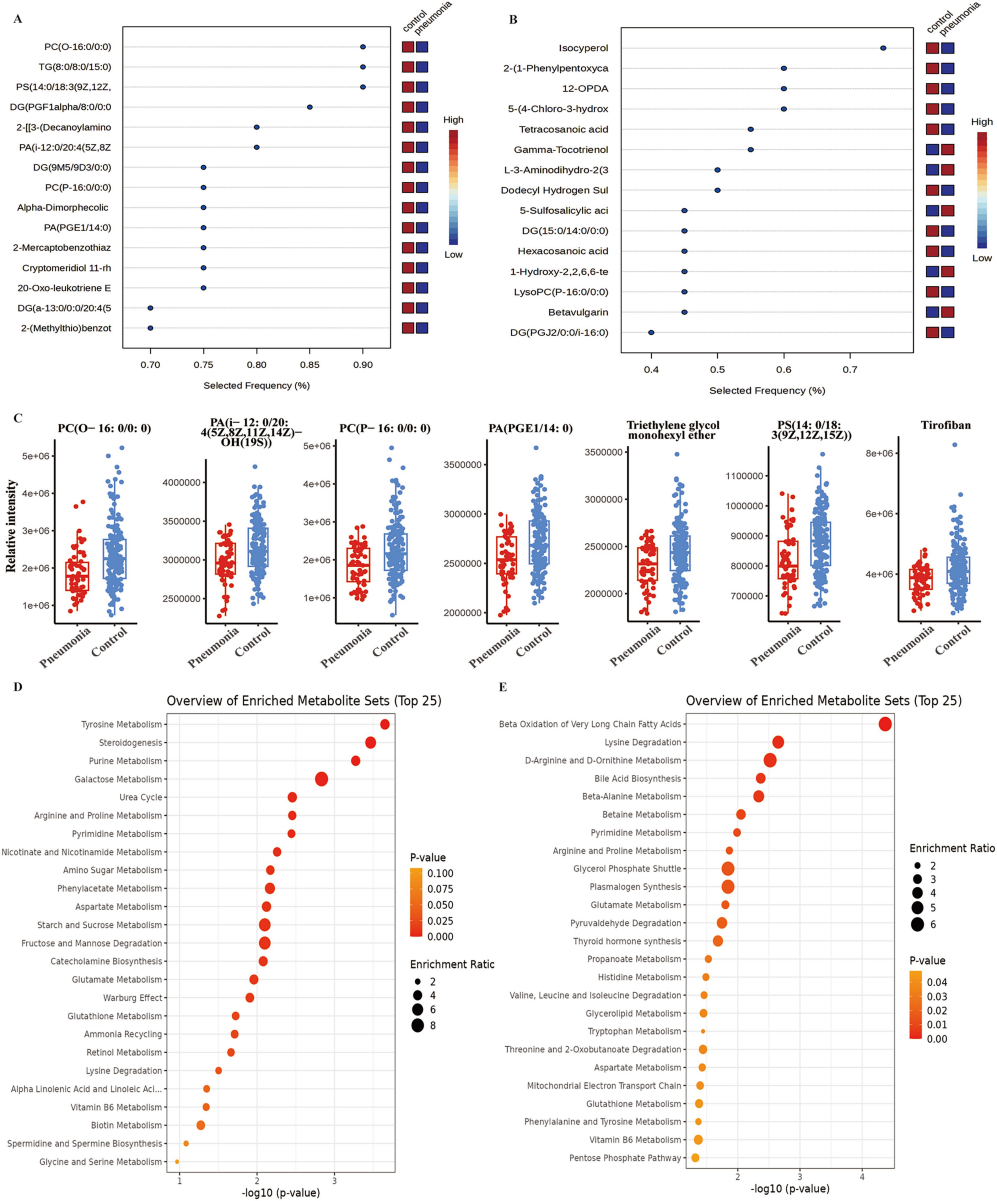
Plasma metabolite biomarkers and pathway enrichment in influenza patients with and without secondary pneumonia. Positive ion mode biomarker analysis results **(A)**. Negative ion mode biomarker analysis results **(B)**. Box plots of up-regulated compounds (Up_compounds) with full metabolite names selected by OPLS-DA model integrating both ion modes **(C)**. SMPDB enrichment dot plot for positive ion mode metabolites **(D)**. SMPDB enrichment dot plot for negative ion mode metabolites **(E)**. Data representation: Box plots in **(C)** show median, interquartile range, and whiskers extending to 1.5× IQR. SMPDB dot plots in **(D,E)** display enrichment ratio versus –log₁₀(*p*-value), with point size proportional to the number of metabolites per pathway.

Dual-ion mode metabolomics identified robust biomarkers that discriminate pneumonia cases from controls. In positive mode, the top 15 discriminative metabolites included PC(O-16:0/0:0), TG(8:0/8:0/15:0), and PS(14:0/18:3(9Z,12Z,15Z)) (Figure 6A); negative mode identified isocyperol, 2-(1-phenylpentoxycarbonyl)benzoic acid, and 12-OPDA (Figure 6B). VIP-selected compounds revealed significant depletion of all 12 top-ranked metabolites in pneumonia cases, including phospholipids (PC(O-16:0/0:0), PC(P-16:0/0:0)), PA(i-12:0/20:4(5Z,8Z.)), PA(PGE1/14:0), triethylene glycol monohexyl ether, PS(14:0/18:3(9Z,12Z,15Z)), and tirofiban (Figure 6C), indicating systemic lipid exhaustion as a hallmark of secondary pneumonia.

Positive-mode metabolites demonstrated superior discriminative performance, with cross-validated PLS-DA achieving AUC = 0.745 (Supplementary Figures S2A,B). Four individual metabolites showed discriminatory capacity: PC(O-16:0/0:0) (AUC = 0.702, 95% CI: 0.626–0.772), TG(8:0/8:0/15:0) (AUC = 0.694, 95% CI: 0.614–0.768), PS(14:0/18:3(9Z,12Z,15Z)) (AUC = 0.696, 95% CI: 0.624–0.765), and DG(PGF1伪/8:0/0:0) (AUC = 0.712, 95% CI: 0.630–0.785) (Supplementary Figures S2C–F), all of which were significantly depleted in pneumonia cases. Negative-mode performance was moderate (PLS-DA AUC = 0.602, 95% CI: 0.48–0.702; Supplementary Figures S3A,B), with top discriminative metabolites including isocyperol (AUC = 0.691), 2-(1-phenylpentoxycarbonyl)benzoic acid (AUC = 0.611), 12-OPDA (AUC = 0.685), and cis-vaccenic acid (AUC = 0.705) (Supplementary Figures S3C–F). These findings establish a plasma metabolomic profile that complements oropharyngeal dysbiosis, with top discriminative metabolites spanning phospholipids, triglycerides, fatty acids, xenobiotics, and oxylipins, indicating broad metabolic reprogramming within this case–control sample.

SMPDB pathway enrichment of positive-mode metabolites revealed significant perturbations in central metabolism, with tyrosine metabolism, steroidogenesis, and purine metabolism showing −log_10_ > 3 and a *p-*value < 0.05 (Figure 6D), representing anabolic processes critical for cell proliferation, signaling, and stress response. Negative-mode metabolites enriched for “beta-oxidation of very long-chain fatty acids” (Figure 6E), indicating catabolic energy metabolism. Collectively, these complementary pathway enrichments highlight a metabolic shift toward catabolic dominance and suppressed anabolism—a profile reminiscent of the Warburg effect and energy crisis in severe infection, where host cells prioritize rapid ATP generation via fatty acid oxidation while inflammatory signaling suppresses biosynthetic pathways.

### 3.6 Multi-omics machine learning identifies *Peptococcus* as an exploratory indicator of secondary pneumonia

**Figure 7:**
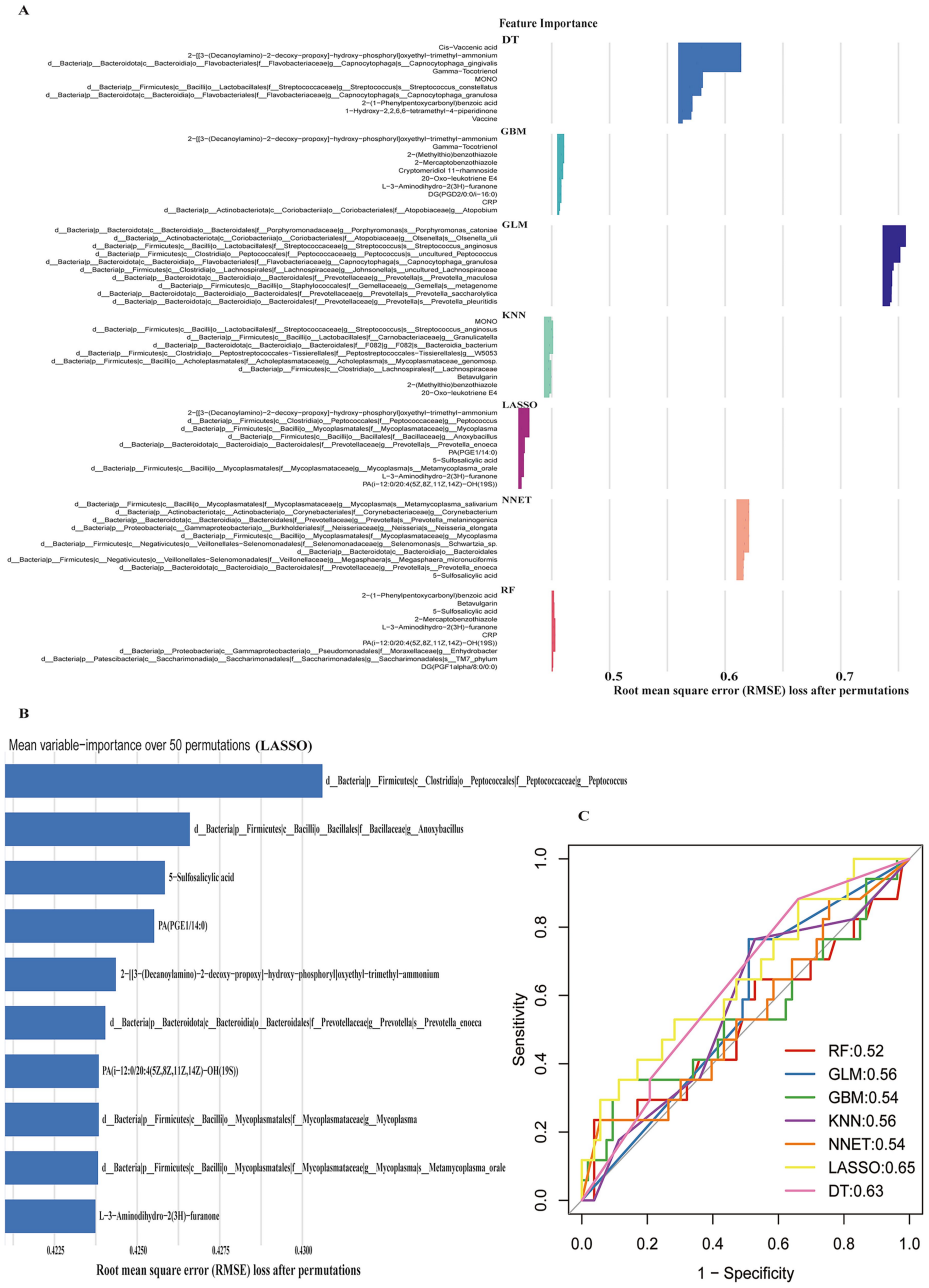
Machine learning model performance and discriminative feature importance for secondary pneumonia in influenza patients. Top 10 feature importance rankings across seven machine learning models (Decision Tree, Gradient Boosting Machine, Generalized Linear Model, K-Nearest Neighbors, Neural Network, Random Forest, and LASSO) **(A)**. Detailed mean variable importance for top seven features in the LASSO model **(B)**. ROC curves comparing discriminateive performance of all seven models **(C)**. Data representation: Feature importance plots **(A,B)** display root mean square error (RMSE) loss after permutations. ROC curves **(C)** show true positive rate versus false positive rate with 95% confidence bands.

To identify integrated biomarkers from the oropharyngeal microbiome, plasma metabolome, and clinical data, we trained seven machine learning algorithms on 236 influenza patients (59 pneumonia cases and 177 controls). Residual analysis, model selection frequency, and precision–recall curves confirmed LASSO’s superior performance (Supplementary Figures S4A–D). Feature importance analysis across models revealed *Peptococcus* as the most discriminative variable, accompanied by *Anoxybacillus*, plasma metabolites (5-sulfosalicylic acid and PA(PGE1/14:0)), and additional taxa (Figure 7B). ROC comparison demonstrated that LASSO achieved optimal discriminative performance (AUC = 0.65), outperforming Random Forest (0.52), Gradient Boosting (0.54), and Neural Network (0.54) (Figure 7C). While this performance is significantly better than chance, we acknowledge that it represents moderate discriminative ability that is insufficient for standalone clinical decision-making. The value of this exploratory analysis lies in identifying biologically plausible multi-omics features rather than providing a validated clinical prediction tool. SHAP analysis revealed a monotonic increase in pneumonia risk with higher *Peptococcus* abundance (Supplementary Figures S4E,F), establishing *Peptococcus* and lipid metabolites as robust, biologically plausible discriminators of secondary pneumonia within this cross-sectional case–control sample.

## 4 Discussion

Secondary bacterial pneumonia is a devastating complication of influenza infection (Bartley et al., 2021; Park et al., 2021; Santus et al., 2024; Lei et al., 2025; Li et al., 2025); however, the biological determinants that characterize uncomplicated influenza versus life-threatening lung infection remain poorly understood. While the gut microbiome is broadly linked to respiratory immunity via the gut–lung axis (Lane et al., 2022; Morgan and Shivanna, 2025), the role of the oropharyngeal microbiome—the microbial community most proximal to the site of secondary infection—has been surprisingly understudied. Our approach aligns with seminal studies (Hu et al., 2016), which demonstrated oropharyngeal dysbiosis in H7N9 influenza patients with secondary bacterial infection and with broader literature using upper respiratory sampling to infer lower tract dynamics. However, we extend these observations by integrating the oropharyngeal microbiome with systemic metabolomics to capture both the local microbial reservoir and host metabolic consequences. By leveraging machine learning to unify microbial, metabolic, and clinical data, we identify *Peptococcus* as a robust, biologically plausible indicator of pneumonia status, establishing a foundation for mechanistic studies and clinical biomarker development.

The oropharyngeal microbiome of pneumonia cases was characterized by enrichment of *Synergistia* and *Bifidobacteriaceae* and depletion of *Bacillaceae*—a compositional shift occurring independently of overall microbial richness. This finding aligns with emerging evidence suggesting that *Synergistia* may exacerbate local inflammation through sulfur-reducing metabolism in inflammatory mucosal environments (Tursi and Papa, 2024). However, our integration of the oropharyngeal microbiome with the plasma metabolome provides novel insights into the systemic consequences of local microbial alterations. The association between pneumonia-enriched taxa and elevated neutrophils and CRP suggests that these microbes may either thrive under hyperinflammatory conditions or actively propagate inflammatory signaling (Morgan and Shivanna, 2025). Conversely, elevated *Bacillaceae* in controls is noteworthy given this family’s known production of antimicrobial peptides and immunomodulatory compounds that may confer colonization resistance against pathogen invasion (Markelova and Chumak, 2025). The dissociation between alpha diversity and disease status underscores a critical principle: In acute infectious contexts, specific taxonomic configurations rather than global diversity metrics drive pathophysiology. This finding mirrors observations in COVID-19 where compositional shifts, not diversity loss, characterized disease severity (Bredon et al., 2025; Jonjić et al., 2025).

Functional inference revealed a clear dichotomy in microbial metabolism between groups. Controls exhibited enrichment of glycolysis, pentose phosphate, and lipid/polysaccharide pathways, indicating a homeostatic state supporting epithelial barrier integrity and immune competence (Hu et al., 2024). Pneumonia cases showed elevated GLUCARDEG degradation, and polyamine synthesis pathways, reflecting a metabolic shift toward nutrient scavenging and stress adaptation (Hu et al., 2024). Strong correlations between *Pseudomonas*, *Achromobacter*, and *Pedobacter* with aromatic compound degradation pathways (GALACTARDEG-PWY and CATECHOL-ORTHO-CLEAVAGE-PWY) suggest the capacity to metabolize host-derived catecholamines and inflammatory mediators, potentially modulating local immune tone (Wang et al., 2024; Lauten et al., 2025). Meanwhile, *Neisseria*’s association with denitrification and FASYN-INITIAL-PWY indicates a microaerophilic niche favoring pathoadaptation (Barth et al., 2009). These taxa–pathway interactions provide a mechanistic scaffold linking oropharyngeal dysbiosis to immunometabolic signaling that may characterize susceptibility to bacterial invasion. We caution against over-interpretation of pathway analyses. PICRUSt2 predictions are based on reference genome databases and may not accurately reflect *in situ* gene expression, particularly for understudied taxa. Similarly, plasma metabolite changes reflect the integrated output of host, microbial, and dietary metabolism and cannot be directly attributed to specific microbial activities without isotope tracing or metabolite source partitioning studies. Our pathway interpretations are intended to generate mechanistic hypotheses for experimental validation rather than definitive functional assignments.

Plasma metabolomics uncovered a surprising profile of systemic lipid depletion rather than the hyperlipidemia typically associated with acute inflammation (Zeng et al., 2024). Phospholipids (PC(O-16:0/0:0), PS(14:0/18:3(9Z,12Z,15Z)), and triethylene glycol monohexyl ether) were significantly depleted in pneumonia cases, forming tightly co-regulated modules that anti-correlated with xenobiotic metabolites and long-chain fatty acids. This pattern suggests metabolic exhaustion, where host lipid reserves are consumed faster than the replenished ones, reminiscent of the Warburg effect and energy crisis observed in severe sepsis (Preau et al., 2021; Vandewalle and Libert, 2022). SMPDB pathway enrichment supported this interpretation: Positive-mode metabolites mapped to suppressed anabolic pathways (tyrosine metabolism, steroidogenesis, and purine metabolism), while negative-mode metabolites enriched for beta-oxidation of very long-chain fatty acids, indicating a shift toward energy extraction through lipid catabolism. Depletion of phospholipids that maintain pulmonary surfactant integrity may directly compromise lung defense (Numata et al., 2009; Barriga et al., 2021), creating a permissive environment for secondary bacterial seeding. We acknowledge that the AUC values (0.694–0.712) indicate moderate discriminative performance, substantially below the >0.90 threshold typically required for clinical biomarker implementation. However, as the first study to integrate oropharyngeal microbiome and plasma metabolome in this clinical context, these results provide important proof-of-concept evidence. The strength of our multi-omics approach lies in identifying biological pathways and generating hypotheses for targeted validation, rather than delivering immediately clinically applicable prediction models.

Machine learning integration identified *Peptococcus* as the paramount indicator of pneumonia status. Its consistent ranking across algorithms, monotonic risk relationship in SHAP analysis, and integration with top-performing metabolites (5-sulfosalicylic acid and PA(PGE1/14:0)) suggest that it sits at the nexus of microbial-metabolic crosstalk. *Peptococcus* is an anaerobic, asaccharolytic genus that ferments amino acids, potentially producing metabolic byproducts that alter local pH (Murdoch, 1998) or reflect overgrowth in hypoxic, inflamed mucosal niches (Litvak et al., 2017). Our multi-omics framework elevates *Peptococcus* from a peripheral commensal to a central indicator of pneumonia status in influenza. We acknowledge that LEfSe and PLS-DA feature filtering were performed on the complete dataset prior to cross-validation, which may introduce optimistic bias. However, the final LASSO regularization step was performed within cross-validation folds, with feature selection repeated in each training split. The L1 penalty provides strong regularization that mitigates overfitting even when initial feature screening was conducted globally. LASSO’s superiority over ensemble methods (Random Forest and Gradient Boosting) likely reflects its capacity to select sparse, biologically meaningful features from high-dimensional omics data, avoiding overfitting to noisy taxonomic variants (Arenas et al., 2021).

Our findings carry immediate translational implications. First, quantitative PCR for *Peptococcus* in oropharyngeal swabs could serve as a rapid, cost-effective screening tool to characterize high-risk influenza patients for enhanced surveillance. Second, the identified metabolic pathways offer therapeutic targets: Lipid supplementation strategies or modulation of polyamine synthesis may restore metabolic homeostasis and attenuate progression. Third, the association between pneumonia-enriched taxa and systemic inflammation suggests that early anti-inflammatory intervention could mitigate dysbiosis-driven susceptibility in similar populations.

Our study has several limitations. The cross-sectional design precludes definitive causal inference; therefore, we cannot determine whether the observed microbiome and metabolome alterations represent causes, consequences, or merely correlates of pneumonia progression. Specifically, the enrichment of *Peptococcus* may reflect pathogenic expansion that promotes pneumonia development, selective growth in anaerobic environments created by pneumonia, or an epiphenomenon of systemic inflammatory status. Similarly, the systemic lipid depletion profile may represent metabolic exhaustion secondary to severe infection rather than a pre-existing susceptibility factor. The modest age difference between groups, although statistically corrected, may influence microbial composition independent of unmeasured severity and treatment differences between groups. Future studies should consider stricter calipers with 1:1 or 1:2 matching to improve balance, age-stratified analyses, or restriction to age-homogeneous subgroups. Our findings are based on single-center data from the 2022–2023 influenza season in Jiangsu Province, China. The identified microbiome-metabolite profile requires validation in diverse geographic settings, different influenza seasons, and varying viral strains to establish generalizability. We explicitly position these findings as preliminary discoveries that require independent replication before clinical translation. We did not apply uniform multiple testing correction across all analyses, and the findings should be considered hypothesis-generating rather than definitive. These findings should be interpreted as hypothesis-generating exploratory results that require validation through longitudinal cohort studies with stricter nested cross-validation or external validation protocols and experimental models to establish causality.

## Data Availability

Publicly available datasets were analyzed in this study. This data can be found here: The 16S rRNA gene sequencing raw sequence reads (fastq) is available in the NCBI Sequence Read Archive under accession projects PRJNA1331894 (https://www.ncbi.nlm.nih.gov/bioproject/PRJNA1331894). Metabolomics data are deposited at MetaboLights: MTBLS13112. No custom code was generated in this paper.

## Glossary

UPLC-MS/MS: Ultra-performance liquid chromatography–tandem mass spectrometry
AUC: Area under the (receiver operating characteristic) curve
SMPDB: Small molecule pathway database
ICU: Intensive care unit
SD: Standard deviation
WBC: White blood cell count
NEU_R: Neutrophil ratio
LYM_R: Lymphocyte ratio
MONO_R: Monocyte ratio
EOS_R: Eosinophil ratio
BAS_R: Basophil ratio
NEU: Absolute neutrophil count
LYM: Absolute lymphocyte count
MONO: Absolute monocyte count
EOS: Absolute eosinophil count
BAS: Absolute basophil count
RBC: Red blood cell count
HB: Hemoglobin
HCT: Hematocrit
MCV: Mean corpuscular volume
MCH: Mean corpuscular hemoglobin
MCHC: Mean corpuscular hemoglobin concentration
RDW: Red cell distribution width
PLT: Platelet count
MPV: Mean platelet volume
PCT: Plateletcrit
PDW: Platelet distribution width
CRP: C-reactive protein
LEfSe: Linear discriminant analysis effect size
LDA: Linear discriminant analysis
RDA: Redundancy analysis
PLS-DA: Partial least squares discriminant analysis
OPLS-DA: Orthogonal PLS-DA
VIP: Variable importance in projection
KEGG: Kyoto encyclopedia of genes and genomes
DT: Decision tree
GBM: Gradient boosting machine
GLM: Generalized linear model
KNN: K-Nearest neighbors
NNET: Neural network
RF: Random forest
