# Supplementary material for: A multi-omics case–control study identifying oropharyngeal microbiome–metabolite patterns that characterize secondary bacterial pneumonia among influenza patients
Source: Front Microbiol. 2026 May 22;17:1824965. doi: 10.3389/fmicb.2026.1824965 (PMC13236517; doi:10.3389/fmicb.2026.1824965)
Supplement: Supplementary file 1 [file Data_Sheet_1.pdf]

Supplementary Material

Contents

Table S1. Demographic and clinical characteristics of influenza patients with and without secondary pneumonia 2

Figure S1. Oropharyngeal microbiome characteristics in influenza patients with and without secondary pneumonia 3

Table S2. Serum biochemical parameters in influenza patients with and without secondary pneumonia 5

Figure S2. Plasma metabolomic biomarkers (positive ion mode) in influenza patients with versus without secondary pneumonia 7

Figure S3. Plasma metabolomic biomarkers (negative ion mode) in influenza patients with versus without secondary pneumonia 9

Figure S4. Machine learning model performance and microbiome feature importance in influenza patients with versus without secondary pneumonia 11

Table S1. Demographic and clinical characteristics of influenza patients with and without secondary pneumonia

characteristic	**Pneumonia(n1=59)**	**Control(n2=177)**	***P***	**Total(N=236)**
Age (years)	45.66±15.70	39.70±17.18	***	41.19±16.98
Male:Female	32:27	98:79	-	130:106
History of influenza vaccination within three years	2(3.39%)	0	-	2(0.85%)
Chronic respiratory system diseases	5(8.47%)	7(3.95%)	-	12(5.08%)
History of respiratory or intestinal surgery	2(3.39%)	7(3.95%)	-	9(3.81%)
Antibiotic use	16(27.12%)	46(25.99%)	-	62(26.27%)
Maximum body temperature (°C)	38.49±0.96	38.52±0.89	-	38.51±0.91
Severe cough, purulent sputum, bloody sputum, or chest pain	7(11.86%)	24(13.56%)	-	31(13.14%)
Tachypnea, dyspnea, cyanosis of the lips	0	6(3.39%)	-	6(2.54%)
Severe vomiting,diarrhea,dehydration	8(13.56%)	34(19.21%)	-	42(17.80%)
Exacerbation of underlying diseases	0	1(0.56%)	-	1(0.42%)

[a]Characteristics with zero sample counts in both patient groups were excluded (specifically, immune-related diseases and altered mental status).

[b]Data are presented as counts (percentages), with rounding to the appropriate decimal places.

[c]The P-value assesses the statistical significance of differences between groups, calculated using a t-test for age and chi-square or Fisher's exact tests for other characteristics. P-values less than 0.001 are denoted by “***”, while those above 0.05 are indicated by “-”.

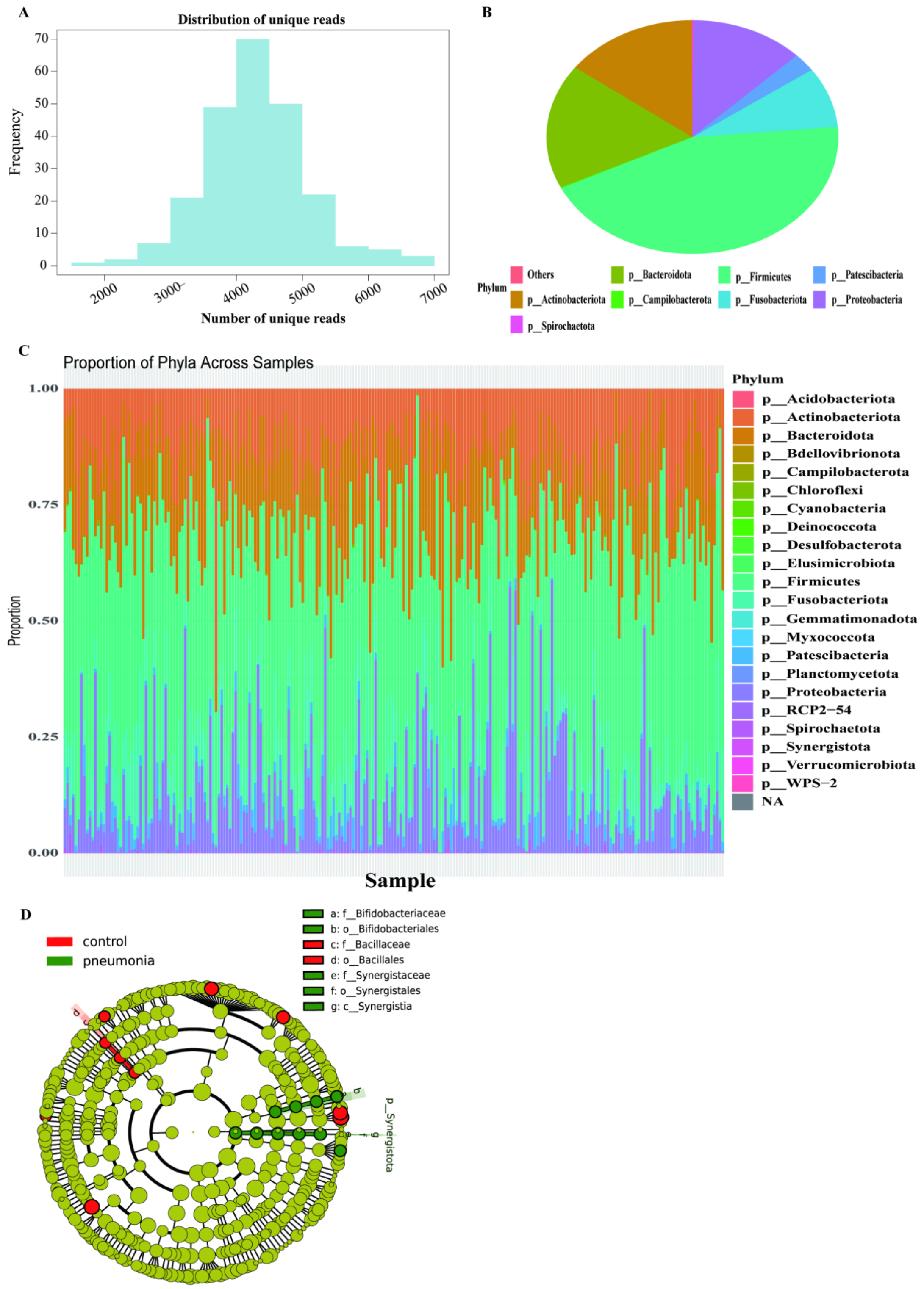

Figure S1. Oropharyngeal microbiome characteristics in influenza patients with and without secondary pneumonia

Histogram of unique sequencing read counts distribution across all samples(A). Aggregate relative

abundance of bacterial phyla pooled across groups(B). Phylum-level taxonomic composition for each individual sample, displayed as stacked bars(C). LEFSE cladogram identifying discriminative taxa between control (influenza alone) and pneumonia (influenza with pneumonia) groups. Green nodes denote taxa enriched in pneumonia; red nodes indicate control-enriched taxa. Circle size corresponds to mean relative abundance(D).Sample size: n = 177 control (influenza) subjects and n = 59 pneumonia (influenza+pneumonia) subjects; full demographics and clinical metadata are provided in Table S1.

Table S2. Serum biochemical parameters in influenza patients with and without secondary pneumonia

parameter	**Pneumonia(n1=59)**	**Control(n2=177)**	***P***	**Total(N=236)**
WBC(10^9/L)	7.56(6.87-8.25)	6.53(6.13-6.93)	0.011(*)	6.79±2.71
NEU_R(%)	75.94(73.51-78.37)	72.81(71.30-74.32)	0.032(*)	73.59±9.97
LYM_R(%)	15.01(13.05-16.96)	16.90(15.66-18.14)	0.109	16.43±8.14
MONO_R(%)	8.35(7.63-9.08)	9.41(8.93-9.90)	0.017(*)	9.15±3.16
EOS_R(%)	0.59(0.32-0.87)	0.72(0.56-0.88)	0.433	0.69±1.07
BAS_R(%)	0.11(0.09-0.13)	0.15(0.13-0.18)	0.004(**)	0.14±0.13
NEU(10^9/L)	5.84(5.22-6.46)	4.88(4.51-5.24)	0.009(**)	5.12±2.47
LYM(10^9/L)	1.06(0.91-1.22)	1.01(0.94-1.08)	0.524	1.02±0.50
MONO(10^9/L)	0.61(0.55-0.67)	0.59(0.55-0.62)	0.603	0.59±0.24
EOS(10^9/L)	0.04(0.02-0.05)	0.04(0.04-0.05)	0.540	0.04±0.06
BAS(10^9/L)	0.007(0.005-0.010)	0.009(0.007-0.010)	0.297	0.008±0.009
RBC(10^12/L)	4.64(4.51-4.77)	4.61(4.54-4.69)	0.721	4.62±4.50
HB(g/L)	143.76(139.91-147.61)	142.83(140.37-145.29)	0.690	143.06±16.03
HCT(%)	0.41(0.40-0.42)	0.41(0.40-0.42)	0.828	0.41±0.05
MCV(fL)	88.09(86.93-89.25)	88.71(88.03-89.38)	0.365	88.55±4.48
MCH(pg)	31.07(30.61-31.53)	30.99(30.70-31.28)	0.774	31.01±1.87
MCHC(g/L)	352.22(350.27-354.18)	349.30(347.78-350.82)	0.021(*)	350.03±9.64
RDW(%)	13.03(12.86-13.20)	13.07(12.93-13.21)	0.699	13.06±0.86
PLT(10^9/L)	175.64(160.84-190.43)	170.71(163.23-178.20)	0.561	171.94±51.67
MPV(fL)	10.20(9.77-10.63)	10.24(10.01-10.46)	0.870	10.23±1.54
PCT(%)	0.17(0.16-0.19)	0.17(0.17-0.18)	0.686	0.17±0.05
PDW(%)	16.34(16.23-16.45)	16.24(16.15-16.33)	0.179	16.26±0.59
CRP(mg/L)	32.98(23.96-42.01)	20.37(16.61-24.13)	0.011(*)	23.52±28.19

[a]Data on pneumonia and influenza are presented as mean ± standard deviation (SD) with 95% confidence intervals (CI), while total data are shown as mean ± SD.

[b]The P-value reflects the statistical significance of differences between groups, with $P < 0.05$ denoted by “*” and $P < 0.01$ by “**” in parentheses.

[c]Values are rounded to two or three decimal places using standard rounding rules.

[d]WBC: White Blood Cell Count; NEU_R: Neutrophil Ratio; LYM_R: Lymphocyte Ratio; MONO_R: Monocyte Ratio; EOS_R: Eosinophil Ratio; BAS_R: Basophil Ratio; NEU: Absolute Neutrophil Count; LYM: Absolute Lymphocyte Count; MONO: Absolute Monocyte Count; EOS: Absolute Eosinophil Count; BAS: Absolute Basophil Count; RBC: Red Blood Cell Count; HB: Hemoglobin; HCT: Hematocrit; MCV: Mean Corpuscular Volume; MCH: Mean Corpuscular Hemoglobin; MCHC: Mean Corpuscular Hemoglobin Concentration; RDW: Red Cell Distribution Width; PLT: Platelet Count; MPV: Mean Platelet Volume; PCT: Platelet Crit; PDW: Platelet Distribution Width; CRP: C-Reactive Protein.

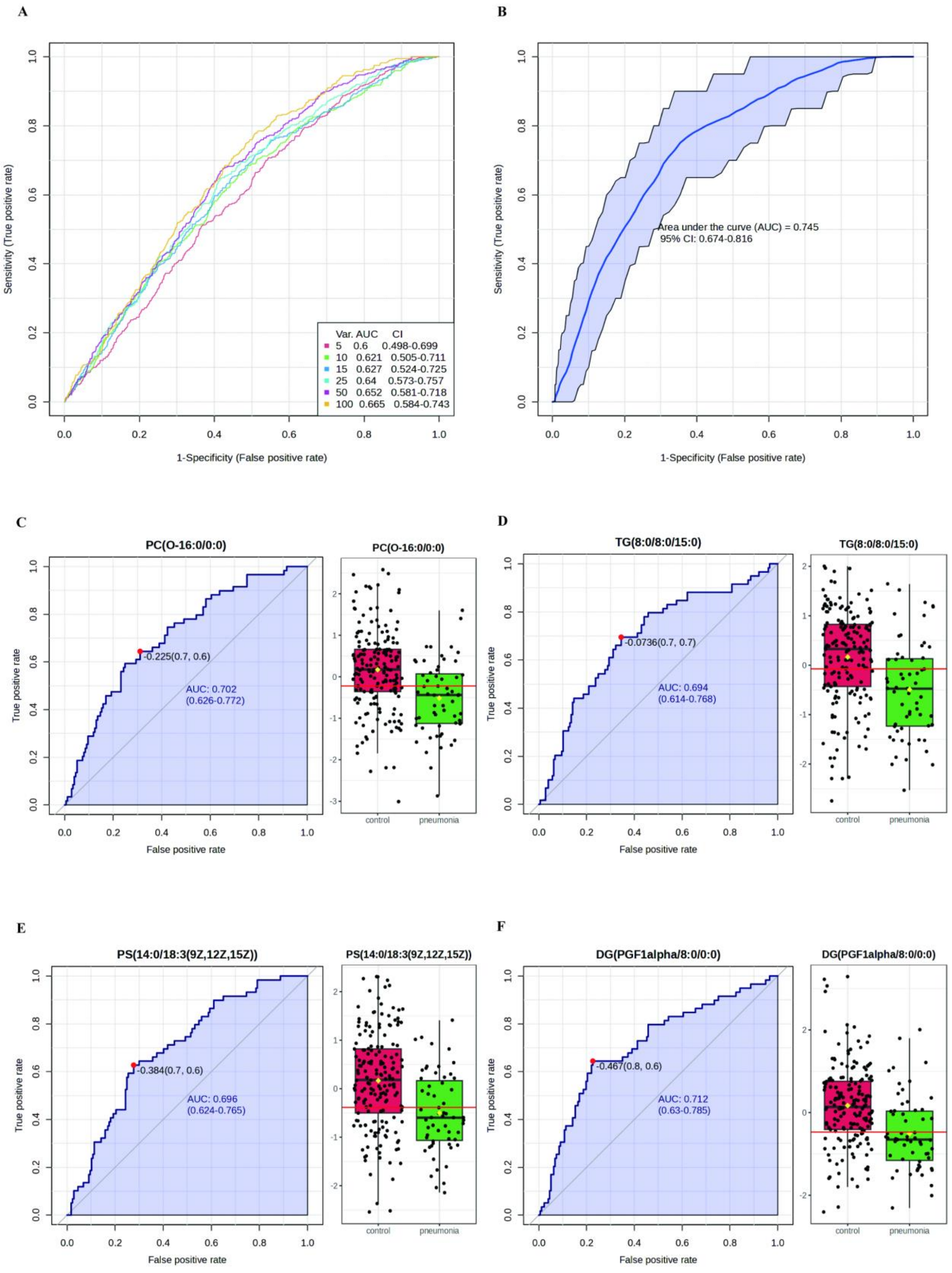

Figure S2. Plasma metabolomic biomarkers (positive ion mode) in influenza patients with versus without secondary pneumonia

ROC curve derived from PLS-DA model trained on positive ion metabolome data (AUC = 0.745, 95% CI: 0.674-0.816)(A) . ROC curve from cross-validated test set predictions(B). Individual ROC curves and abundance box plots for top discriminant metabolites(C – F) : PC(O-16:0/0:0)

(AUC = 0.702, 95% CI: 0.626-0.772)(C), TG(8:0/8:0/15:0) (AUC = 0.694, 95% CI: 0.614-0.768)(D) , PS(14:0/18:3(9Z,12Z,15Z)) (AUC = 0.696, 95% CI: 0.624-0.765) (E), and DG(PGF1alpha/8:0/0:0) (AUC = 0.712, 95% CI: 0.630-0.785)(F) . *n* = 59 influenza with secondary pneumonia and *n* = 177 influenza-only controls; full clinical metadata are provided in Table S1.

Significance: for univariate ROC analyses, only metabolites with AUC > 0.65 and $p < 0.05$ are displayed.

Data representation: Box plots show median, interquartile range, and whiskers extending to 1.5× IQR; ROC plots display true positive rate versus false positive rate.

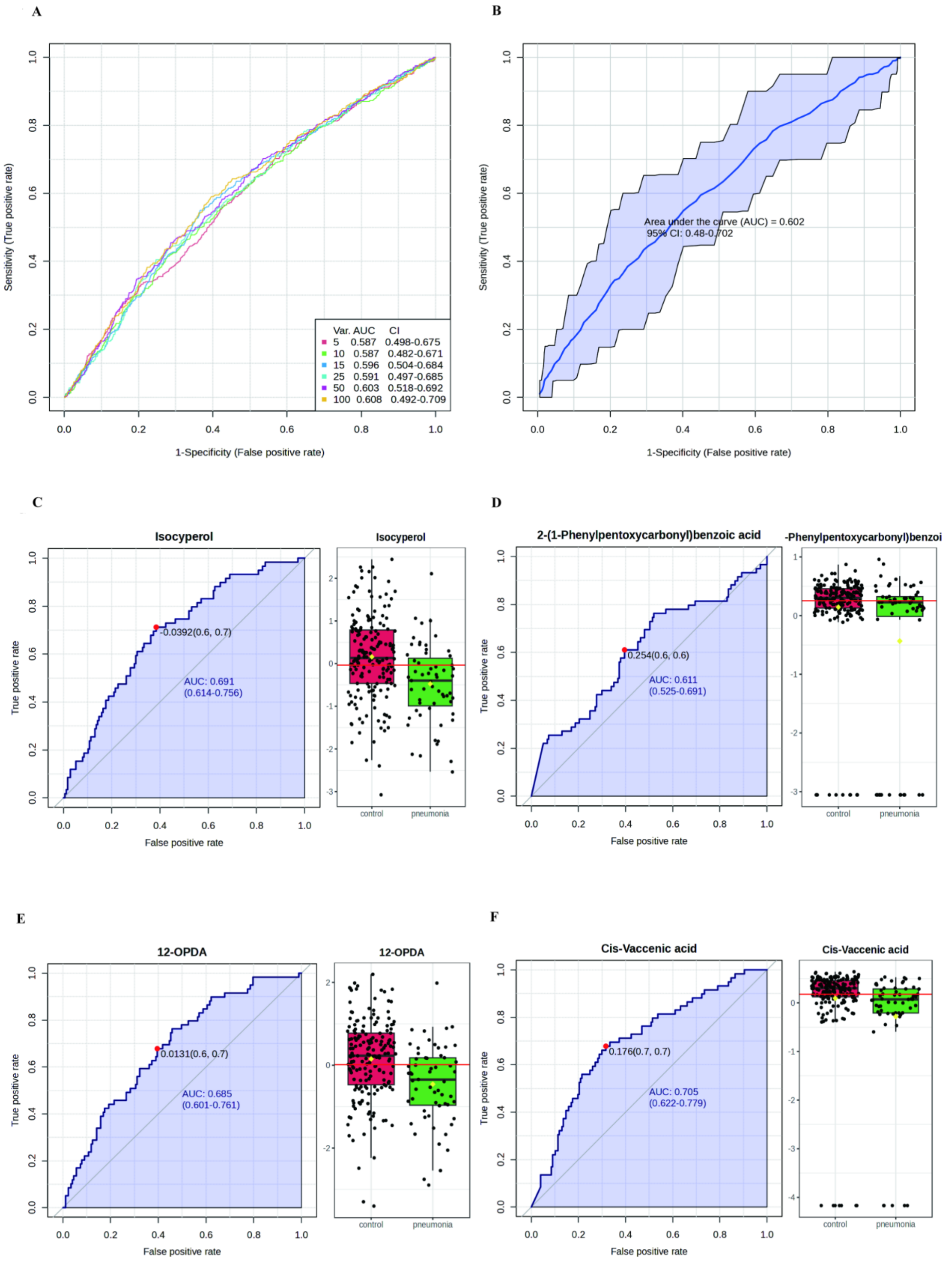

Figure S3. Plasma metabolomic biomarkers (negative ion mode) in influenza patients with versus without secondary pneumonia

ROC curve from PLS-DA classifier trained on negative ion mode metabolome data(A) . Test set ROC performance across varying model component numbers showing AUC values and 95% CIs (AUC = 0.602, 95% CI: 0.48 – 0.702)(B). Univariate ROC curves and abundance box plots for top

discriminant metabolites(C – E) : Isocyperol (AUC = 0.691, 95% CI: 0.614 – 0.756)(C) , 2-(1-Phenylpentoxycarbonyl)benzoic acid (AUC = 0.611, 95% CI: 0.525 – 0.691)(D) , 12-OPDA (AUC = 0.685, 95% CI: 0.601 – 0.761)(E). Cis-Vaccenic acid (AUC = 0.705, 95% CI: 0.622 – 0.779). ROC curve and abundance box plot for Cis-Vaccenic acid, the metabolite with highest individual AUC(F) . *n* = 59 influenza patients with secondary pneumonia and *n* = 177 influenza-only controls; full demographic and clinical characteristics are provided in Table S1.

Significance: for univariate ROC analyses, only metabolites with AUC > 0.65 and $p < 0.05$ are displayed.

Data representation: Box plots show median, interquartile range, and whiskers extending to 1.5× IQR; ROC plots display true positive rate versus false positive rate.

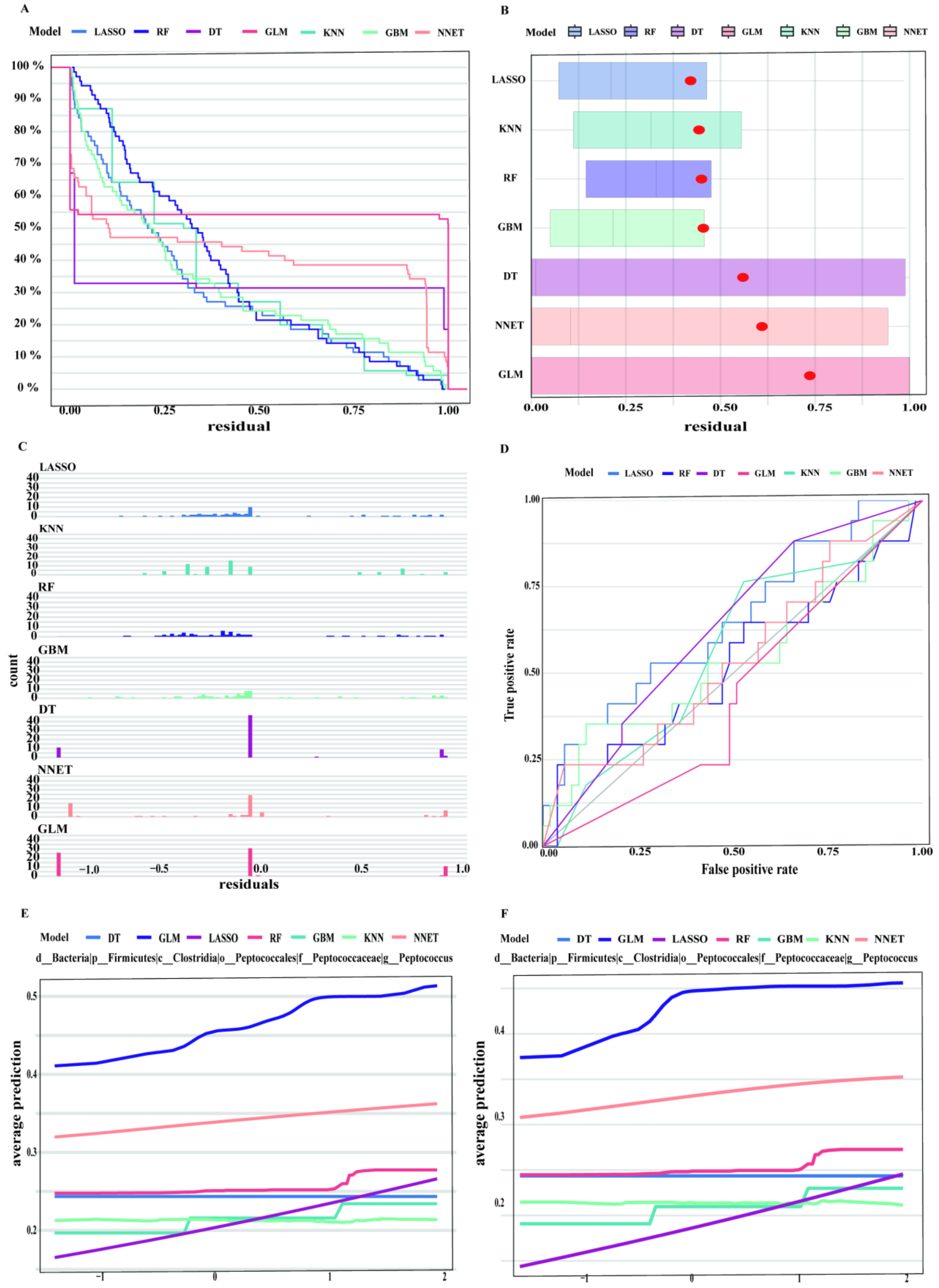

Figure S4. Machine learning model performance and microbiome feature importance in influenza patients with versus without secondary pneumonia

Cumulative distribution of residuals for each model(A) . Residual box plots across seven machine learning models(B) . Model selection frequency histogram(C) . Precision-recall curves for

LASSO, Decision Tree (DT), Generalized Linear Model (GLM), K-Nearest Neighbors (KNN), Random Forest (RF), Gradient Boosting Machine (GBM), and Neural Network (NNET)(D) . SHAP dependence plot for *Peptococcus* (d__Bacteria|p__Firmicutes|c__Clostridia|o__Peptococcales|f__Peptococcaceae|g__Peptococcus)(E) . Cumulative effect plot for Peptococcus on model predictions(F) .

Significance: Model performance was evaluated using mean AUC and precision-recall AUC; only models with mean AUC > 0.60 are shown. Features with mean SHAP values > 0.01 and $p < 0.05$ are displayed.

Data representation: Box plots show median, interquartile range, and whiskers extending to 1.5× IQR. SHAP plots illustrate contribution to log-odds prediction. Histogram bars represent relative model importance normalized to 100%.
